# An upstream enhancer and MEF2 transcription factors fine-tune the regulation of the *Bdnf* gene in cortical and hippocampal neurons

**DOI:** 10.1016/j.jbc.2024.107411

**Published:** 2024-05-23

**Authors:** Annela Avarlaid, Kaisa Falkenberg, Karin Lehe, Giuseppa Mudò, Natale Belluardo, Valentina Di Liberto, Monica Frinchi, Jürgen Tuvikene, Tõnis Timmusk

**Affiliations:** 1 Department of Chemistry and Biotechnology, Tallinn University of Technology, Tallinn, Estonia; 2 Department of Biomedicine, Neuroscience and Advanced Diagnostic, University of Palermo, Palermo, Italy; 3 Protobios LLC, Tallinn, Estonia

**Keywords:** brain-derived neurotrophic factor, BDNF, myocyte enhancer factor 2, MEF2, enhancer, hippocampus, cortex, transcriptional regulation

## Abstract

The myocyte enhancer factor (MEF2) family of transcription factors, originally discovered for its pivotal role in muscle development and function, has emerged as an essential regulator in various aspects of brain development and neuronal plasticity. The MEF2 transcription factors are known to regulate numerous important genes in the nervous system, including brain-derived neurotrophic factor (BDNF), a small secreted neurotrophin responsible for promoting the survival, growth, and differentiation of neurons. The expression of the *Bdnf* gene is spatiotemporally controlled by various transcription factors binding to both its proximal and distal regulatory regions. While previous studies have investigated the connection between MEF2 transcription factors and *Bdnf*, the endogenous function of MEF2 factors in the transcriptional regulation of *Bdnf* remains largely unknown. Here, we aimed to deepen the knowledge of MEF2 transcription factors and their role in the regulation of *Bdnf* comparatively in rat cortical and hippocampal neurons. As a result, we demonstrate that the MEF2 transcription factor-dependent enhancer located at −4.8 kb from the *Bdnf* gene regulates the endogenous expression of *Bdnf* in hippocampal neurons. In addition, we confirm neuronal activity-dependent activation of the −4.8 kb enhancer *in vivo*. Finally, we show that specific MEF2 family transcription factors have unique roles in the regulation of *Bdnf,* with the specific function varying based on the particular brain region and stimuli. Altogether, we present MEF2 family transcription factors as crucial regulators of *Bdnf* expression, fine-tuning *Bdnf* expression through both distal and proximal regulatory regions.

Myocyte enhancer factor 2 (MEF2) transcription factors belong to the MADS box transcription factor superfamily, which in vertebrates consists of four paralogous members (MEF2A-D) (1, 2, 3). While the initial identification of MEF2 factors was predominantly associated with myogenesis (4, 5), subsequent research has demonstrated their pleiotropic roles within the central nervous system, where MEF2A, MEF2C, and MEF2D are mainly expressed (3, 6). It has become increasingly evident that MEF2 transcription factors have a significant role in synaptogenesis, neuronal survival, differentiation, and plasticity (7). All MEF2 proteins have a highly conserved N-terminus, containing the MADS-box and MEF2 domain, and a divergent C-terminus important for transactivation. The MADS-box and MEF2 domains mediate dimerization and binding of the MEF2 homo- or heterodimers to DNA consensus site (C/T)TA(A/T)_4_TA(G/A), known as MEF2 response element (MRE) (8, 9, 10). After binding, MEF2 transcription factors can activate or repress transcription of their target genes by recruiting coregulators like histone acetyltransferases or deacetylases, respectively (11, 12, 13, 14).

One of the target genes of MEF2 transcription factors is *Bdnf* (6, 10, 15, 16), a neurotrophin that has an important role in the developing and mature organism by modulating neuronal survival, development, and plasticity (17). Rodent *Bdnf* gene contains eight 5′ non-coding exons (I-VIII) and one 3′ protein-coding exon (IX), with each exon regulated by its distinct promoter (18). The *Bdnf* transcripts are generated by splicing one of the non-coding exons (I-VIII) together with the coding exon (IX) (18, 19). In general, *Bdnf* transcripts of the first cluster (exon I, II, III) have a nervous system-specific expression pattern, whereas *Bdnf* exon IV- and VI-containing transcripts are expressed in both neural and non-neural tissues (18, 19, 20). The spatiotemporal expression of *Bdnf* gene is ensured by various transcription factors binding to its distinct promoters (16, 21, 22, 23) and through the use of different enhancer regions (24, 25, 26).

The role of MEF2 family transcription factors in the regulation and signaling of brain-derived neurotrophic factor (BDNF) has been previously demonstrated in numerous studies (6, 10, 15, 21, 27). For example, the BDNF-activated ERK5-MEF2 signaling pathway has been shown to induce the survival of newly generated cerebellar granule neurons (27) and embryonic cerebral neurons (28). Also, silencing of *Mef2a* and *Mef2d* using RNA interference reduced activity-dependent induction of *Bdnf* in rat hippocampal neurons (10). In rat cortical neurons, knockdown of *Mef2d* strongly increased activity-dependent expression of the *Bdnf* exon I-containing transcripts, while knockdown of *Mef2a* or *Mef2c* reduced neuronal activity-dependent expression of *Bdnf* exon I- or *Bdnf* exon IV-containing transcripts, respectively (6). Moreover, it has been shown that MEF2D needs the presence of CREB to bind *Bdnf* promoter IV in mouse cortical neurons (15), and we have previously shown the binding of MEF2A and MEF2C to *Bdnf* promoter IV by *in vitro* DNA pulldown assay in rat cortical tissue nuclear lysates (16). Finally, a region ∼4.8 kb upstream of the *Bdnf* promoter I encompasses MRE, binds MEF2D transcription factor, and has been shown to regulate the neuronal activity-dependent transcription of *Bdnf* promoter I-driven reporter constructs in hippocampal neurons (10) but not in cortical neurons (6).

Although the role of MEF2 in the regulation of *Bdnf* has been described in several previous studies, the endogenous activity of the −4.8 kb MEF2-dependent enhancer and the contribution of MEF2 family transcription factors to the major *Bdnf* transcripts has not been addressed. Here, we aimed to comparatively study the role of MEF2 transcription factors in the regulation of *Bdnf* expression in cortical and hippocampal neurons. To achieve this, we used CRISPR/dCas9 system to show for the first time that the −4.8 kb MEF2 transcription factor-dependent enhancer regulates the endogenous expression of the first cluster of *Bdnf* transcripts in cultured hippocampal neurons. To elucidate the activity of the −4.8 kb enhancer *in vivo*, we used kainic acid-injected animals and detected robust activity-dependent transcription of −4.8 kb eRNAs in the hippocampus. Finally, RNA interference-mediated knockdown of MEF2 family members in both cultured cortical and hippocampal neurons revealed an interplay between MEF2 transcription factors in the regulation of *Bdnf* expression through promoter regions and the −4.8 kb enhancer. Together, these findings demonstrate how various members of the MEF2 family of transcription factors exert distinct effects on the expression of *Bdnf* depending on stimuli and brain regions.

## Results

### The enhancer located 4.8 kb upstream of *Bdnf* exhibits neuronal activity-dependent transcription

**Figure 1:**
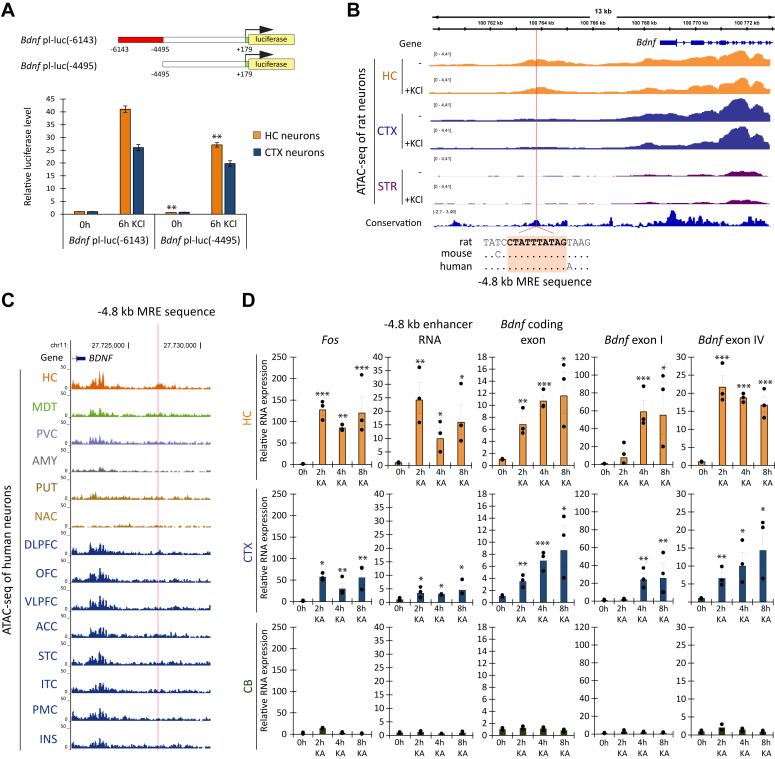
**The −4.8 kb enhancer exhibits enhancer-related characteristics in hippocampal neurons and adult hippocampal tissue.***A*, schematics of luciferase constructs used for transfection. *Red* box represents mouse −4.8 kb enhancer region, *white* box mouse genomic region upstream of *Bdnf* promoter I, and mouse *Bdnf* promoter I (*arrow* indicates TSS) and *yellow* box indicates luciferase coding region. At 5 DIV, rat primary cortical and hippocampal neurons were transfected with the indicated constructs. At 6 DIV, tetrodotoxin (TTX) was added to neurons to inhibit spontaneous neuronal activity. At 7 DIV, transfected neurons were treated with KCl (25 mM) and D-APV (5 μM) for 6 h or *left* untreated (CTRL). *Bdnf* promoter I activity was measured using luciferase assay. Luciferase levels measured from rat cortical or hippocampal neurons transfected with *Bdnf* pI-luc(-6143) and *left* untreated were taken as 1. Error bars indicate SEM (n = 4). For statistical analysis, two-tailed paired *t* test was preformed between the luciferase levels in cells transfected with Bdnf pI-luc(-6143) and *Bdnf* pI-luc(-4495) upon the respective treatment. *B*, the Integrative Genomics Viewer tool was used to characterize the −4.8 kb enhancer region based on previously conducted ATAC-seq data (29), where rat cultured hippocampal, cortical and striatal neurons were treated with 10 mM KCl (1 h KCl) or vehicle (Neurobasal medium). The first cluster of rat *Bdnf* exons is shown as *blue* boxes (exons) and horizontal lines connecting boxes (introns). *Orange*-colored tracks represent data from hippocampal neurons (HC), *blue*-colored tracks indicate data from cortical neurons (CTX) and *purple*-colored tracks show data from striatal (STR) neurons. Light *red* vertical line represents the MRE-sequence within the −4.8 kb enhancer. The conservation track shows conservation across 100 vertebrates (phyloP) and the genomic alignment of the −4.8 kb MRE sequence (CTATTTATAG) in rat, mouse and human is indicated by light *red* shading. *C*, UCSC Genome Browser tool (https://genome.ucsc.edu/) was used to describe the −4.8 kb enhancer region based on previously conducted (31) NeuN+ ATAC-seq data in different human brain regions: (HC) hippocampus; (MDT) mediodorsal thalamus; (PVC) primary visual cortex; (AMY) amygdala; (PUT) putamen; (NAC) nucleus accumbens; (DLPFC) dorsolateral prefrontal cortex; (OFC) orbitofrontal cortex; (VLPFC) ventrolateral prefrontal cortex; (ACC) anterior cingulate cortex; (STC) superior temporal cortex; (ITC) inferior temporal cortex; (PMC) primary motor cortex; (INS) insula. The first exon (exon I) of human *BDNF* is shown as *blue* box, light *red* vertical line represents the MRE-sequence 4.8 kb upstream of *BDNF* exon I. *D*, Adult (3 months old) male Wistar rats were used for intracerebroventricular injection of kainic acid (KA). The experimental animals were randomly divided into four groups: ICV-injected with saline solution (CTRL) and ICV-injected with KA and sacrificed at the indicated time after the onset of seizures (2 h, 4 h or 8 h kA). The mRNA levels of *Fos*, −4.8 kb enhancer RNA, total *Bdnf* and *Bdnf* exon I- and IV-containing transcripts were measured from the rat hippocampus (HC), cerebral cortex (CTX) and cerebellum (CB). The expression levels were measured using RT-qPCR and normalized to *Hprt1* mRNA levels. The results from individual animals are depicted with dots and the average of respective enhancer RNA or mRNA levels in two to three animals is shown with the column. The average level of respective transcript measured from the CTRL group was set as 1. Error bars indicate SEM (n = 2–3 animals). Statistical significance was calculated with two-tailed unpaired unequal variance *t* test relative to the expression level of the respective transcript levels in the control animals. (A, D) ∗*p* < 0.05, ∗∗*p* < 0.01, ∗∗∗*p* < 0.001.

Previous studies have demonstrated that a putative enhancer located ∼4.8 kb upstream of the *Bdnf* exon I transcription start site promotes neuronal activity-dependent transcription from *Bdnf* promoter I in transient expression assays in hippocampal (10) but not in cortical neurons (6). Since different laboratories conducted these experiments using distinct protocols, we decided to comparatively investigate the impact of −4.8 kb enhancer on *Bdnf* promoter I activity in both cultured rat hippocampal and cortical neurons. For that, we conducted luciferase reporter assays using reporter constructs that were used previously (6, 10) and treated the neurons for 6 h with KCl to mimic neuronal activity. Our results showed that luciferase activity controlled by *Bdnf* promoter I without the −4.8 kb enhancer region decreased ∼40% (*p* = 0.00153) in untreated and ∼34% (*p* = 0.00712) in KCl-treated hippocampal neurons compared to luciferase activity driven by *Bdnf* promoter I with the −4.8 kb enhancer region (Fig. 1*A*). Notably, this effect was less evident and statistically not significant in untreated and KCl-treated cortical neurons, where the luciferase activity driven by *Bdnf* promoter I decreased ∼20% (*p* = 0.09951) and ∼24% (*p* = 0,07329) without the −4.8 kb enhancer, respectively (Fig. 1*A*). These results are in good agreement with previous findings (6, 10), and indicate that in heterologous context the −4.8 kb enhancer region potentiates the basal and KCl-induced activity of *Bdnf* promoter I specifically in hippocampal neurons.

Until now, research focusing on the −4.8 kb enhancer has been limited to luciferase assays. To study the −4.8 kb region as an enhancer in endogenous context, we initially analyzed previously obtained data of assay for transposase-accessible chromatin using sequencing (ATAC-seq) in rat hippocampal, cortical and striatal neurons (29) to study the chromatin state within the −4.8 kb region (Fig. 1*B*). The position of the −4.8 kb enhancer was determined by the MRE sequence previously described in Flavell *et al.* 2008 (10) and located at the center of the enhancer. The −4.8 kb enhancer exhibited stronger ATAC-seq signals in both untreated and KCl-treated rat hippocampal neurons than in cortical neurons. In striatal neurons the ATAC-seq signals within the *Bdnf* and −4.8 kb region were undetectable, which aligns with previous findings demonstrating that the mRNA levels of *Bdnf* are not detectable in rat striatum (Fig. 1*B*) (20, 30). As described previously (10), the −4.8 kb region is highly conserved in vertebrates and the −4.8 kb MRE element has identical sequence among rat, mouse and human, suggesting functionality of the −4.8 kb region also in other vertebrates (Fig. 1*B*). Next, to determine whether the −4.8 kb enhancer could be a regulatory region in humans, we used published ATAC-seq conducted in neurons from different human brain regions (31). Consistent with the ATAC-seq findings from rat neurons, the −4.8 kb region revealed open chromatin, particularly in human hippocampal neurons (Fig. 1*C*).

While open chromatin is widely associated with regulatory elements, it does not indicate a functional enhancer by default. In addition to chromatin accessibility, most of the active enhancers require the binding of RNA polymerase and transcription factors, which leads to the transcription of non-coding enhancer RNAs (eRNAs) (32, 33). It has been previously shown that eRNA levels correlate with the target gene expression (29, 33) and that the expression of *Bdnf* is robustly induced *in vivo* upon kainic acid (KA) treatment in different brain regions (30). Therefore, we hypothesized that −4.8 kb enhancer RNAs are also induced *in vivo* upon KA-induced neuronal activation. To test this hypothesis, rats were injected intracerebroventrically (ICV) with KA, a glutamate receptor agonist used as a robust model of epileptic seizures (34), or saline solution as a control. Rats were sacrificed 2 h, 4 h or 8 h after the onset of epileptic seizures and −4.8 kb eRNA together with *Bdnf* mRNA levels were analyzed using RT-qPCR from hippocampus, cerebral cortex and cerebellum, a brain region where *Bdnf* levels are not induced after KA treatment (30). As a positive control of a neuronal activity regulated gene expression (35, 36), we measured the levels of *Fos* mRNA, that were induced after KA treatment *in vivo* in all analyzed brain regions (Fig. 1*D*). In addition, a strong, up to ∼24-fold upregulation for −4.8 kb eRNAs was seen in rat hippocampus and ∼3-5-fold induction in cerebral cortex, whereas in cerebellum the −4.8 kb eRNA levels were not induced upon KA treatment. Similarly, the mRNA levels of total *Bdnf, Bdnf* exon I and *Bdnf* exon IV were induced in the hippocampus and cortex but not in the cerebellum (Fig. 1*D*). Altogether, these results demonstrate that the −4.8 kb region exhibits enhancer-related characteristics, such as open chromatin and stimulus-dependent transcription, particularly in the hippocampus.

### CRISPR/dCas9 mediated targeting of the −4.8 kb region unveils a hippocampus-specific enhancer of the *Bdnf* gene

**Figure 2:**
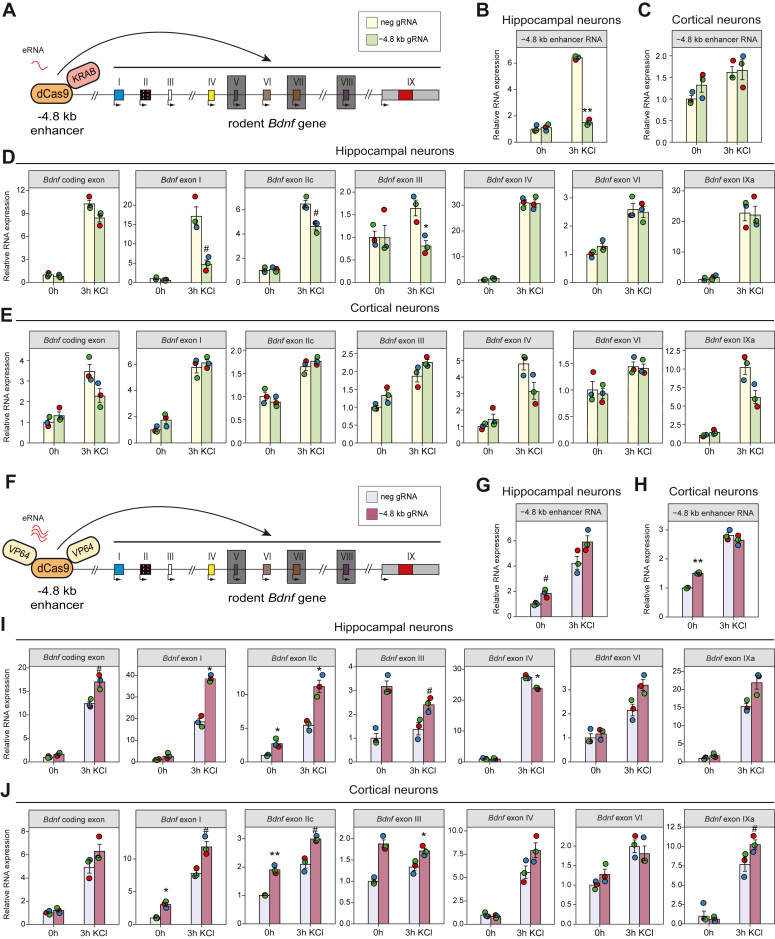
**Targeting the −4.8 kb enhancer with CRISPR/dCas9 system affects the expression of endogenous *Bdnf* in rat hippocampal and cortical neurons.** (*A*–*J*) At 0 DIV, rat hippocampal (*B*, *D*, *G*, *I*) and cortical neurons (*C*, *E*, *H*, *J*) were infected with negative control gRNA (neg gRNA) or with the pool of 3 gRNAs covering the −4.8 kb enhancer region (−4.8 kb gRNA) and dCas9-KRAB (*A*–*E*) or VP64-dCas9-VP64 (*F*–*J*) coding lentiviruses. At 7 DIV, tetrodotoxin (TTX) was added to neurons to inhibit spontaneous neuronal activity. At 8 DIV, neurons were treated with KCl (25 mM) and D-APV (5 μM) for 3 h or *left* untreated. Expression of the −4.8 kb eRNAs (*B*–*C*, *G*–*H*), total *Bdnf* (*Bdnf* coding exon) and different *Bdnf* transcripts (*D*–*E*, *I*–*J*) were measured using RT-qPCR. For every experiment, the expression level of the respective RNA in cells transduced with dCas9-KRAB or VP64-dCas9-VP64 and negative gRNA-coding lentiviruses and *left* untreated was taken as one. The dots indicate results from biological replicates (n = 3), with error bars showing SEM. For statistical analysis, two-tailed paired *t* test was performed between the RNA levels in cells transduced with lentiviruses encoding −4.8 kb enhancer-specific gRNAs and negative control gRNA and either dCas9-KRAB or VP64-dCas9-VP64 upon the respective treatment, #*p* < 0.1, ∗*p* < 0.05, ∗∗*p* < 0.01, ∗∗∗*p* < 0.001.

Enhancers can interact with and regulate one or several promoters located at considerable distances from their genomic location (37, 38). The −4.8 kb enhancer has been associated with the nearest regulatory region, *Bdnf* promoter I, in heterologous context (6, 10). Since the regulation of *Bdnf* expression involves several distinct promoters acting as independent regulatory hubs (18, 30), it is crucial to clarify the function of enhancer with the respect to each *Bdnf* promoter. Additionally, it is important to establish the connection between the enhancer and its target gene in the endogenous context. To study whether the alteration of the −4.8 kb enhancer affects the levels of different transcripts of *Bdnf* in an endogenous context, we used lentivirus-mediated CRISPR/dCas9 system to either repress (Fig. 2*A*) or activate (Fig. 2*F*) the enhancer in rat primary hippocampal and cortical neurons. Cultured neurons were treated for 3 h with KCl to mimic neuronal activity, and the levels of the −4.8 kb enhancer RNA and the *Bdnf* mRNAs were measured using RT-qPCR.

In cultured neurons the expression of −4.8 kb eRNA increased ∼4-6-fold in hippocampal (Fig. 2, *B* and *G*) and ∼2-3-fold in cortical neurons (Fig. 2, *C* and *H*) upon KCl treatment, showing the −4.8 kb region as an activity-dependent enhancer *in vitro*. To repress the enhancer, we used catalytically inactive Cas9 (dCas9) fused with the Krüppel associated box (KRAB) domain (dCas9-KRAB) coupled with 3 distinct gRNAs covering the −4.8 kb enhancer region. Repression of the −4.8 kb enhancer diminished the KCl-dependent expression of −4.8 kb eRNAs in hippocampal (Fig. 2*B*) but not in cortical neurons (Fig. 2*C*). Similarly, the repression of the −4.8 kb enhancer decreased the levels of the first cluster of *Bdnf* transcripts (*Bdnf* exon I-, IIc- and III-containing transcripts) in hippocampal neurons, where the strongest ∼4-fold decrease was observed for *Bdnf* exon I-containing transcripts after KCl treatment (Fig. 2*D*). Repression of the enhancer neither affected the other studied transcripts of *Bdnf* (*Bdnf* exon IV-, VI- and IX-containing transcripts) in hippocampal neurons (Fig. 2*D*) nor the expression levels of *Bdnf* mRNAs in cortical neurons (Fig. 2*E*).

Next, we used tandem repeats of transactivator VP16 domain fused with dCas9 (VP64-dCas9-VP64) together with 3 different gRNAs covering the −4.8 kb enhancer to induce the transcription from the enhancer. The activation of the −4.8 kb enhancer increased the −4.8 kb eRNA levels in untreated hippocampal (Fig. 2*G*) and cortical neurons (Fig. 2*H*). Activation of the −4.8 kb enhancer also increased *Bdnf* levels in untreated and KCl-treated hippocampal (Fig. 2*I*) and cortical neurons (Fig. 2*J*), where the first cluster of *Bdnf* transcripts was upregulated ∼2-3-fold, indicating that the transcriptional activation by the CRISPR activator complex artificially induced the transcription from the −4.8 kb enhancer and upregulation of *Bdnf* expression. Although the −4.8 kb eRNA levels increased in both KCl-treated cultured cortical neurons (Fig. 2, *C* and *H*) and after KA treatment in adult cortical tissue (Fig. 1*D*), the repression of −4.8 kb enhancer had no effect on the −4.8 eRNA (Fig. 2*C*) and *Bdnf* levels in cultured cortical neurons (Fig. 2*E*). Therefore, the role of the −4.8 kb enhancer in the regulation of *Bdnf* in cortical neurons remains to be elucidated. We cannot rule out that the −4.8 kb enhancer regulates *Bdnf* expression in specific cortical cell type(s) or depending on developmental stage. Collectively, here we demonstrate for the first time that in the endogenous context the region 4.8 kb upstream of *Bdnf* is crucial for enhancing the neuronal activity dependent *Bdnf* expression in hippocampal neurons.

### The MEF2 family transcription factors regulate *Bdnf via* both proximal and distal regulatory regions

**Figure 3:**
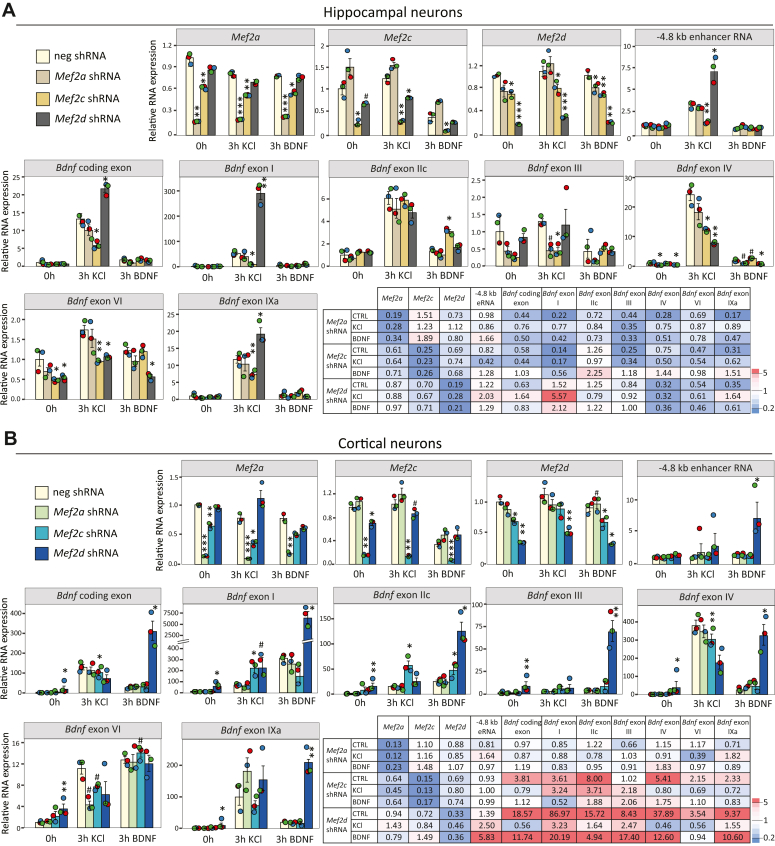
**Silencing of *Mef2* transcription factors alters the transcription from different *Bdnf* promoters and −4.8 kb enhancer in cortical and hippocampal neurons.***A* and *B*, at 0 DIV rat hippocampal (*A*) or cortical (*B*) neurons were infected with lentiviruses encoding *Mef2* shRNAs (*Mef2a, Mef2c* or *Mef2d*) or negative control shRNA (neg shRNA). At 7 DIV, tetrodotoxin (TTX) was added to neurons to inhibit spontaneous neuronal activity. At 8 DIV neurons were treated with 25 mM KCl together with 5 μM D-APV or 50 ng/ml BDNF for 3 h or left untreated (0 h). The mRNA expression levels of different *Mef2* family members, *Bdnf* transcripts and −4,8 kb eRNA were measured using RT-qPCR. The expression levels in cells transduced with negative shRNA-encoding lentiviruses and left untreated was set as 1. All biological replicates (n = 3) are shown as dots. Error bars indicate SEM. Asterisks indicate statistical significance calculated using two-tailed paired *t* test between the respective transcript levels measured from neurons transduced with neg shRNA and indicated *Mef2* shRNA at respective treatment, #*p* < 0.1, ∗*p* < 0.05, ∗∗*p* < 0.01, ∗∗∗*p* < 0.001. To simplify the understanding of the results, we arranged the heatmap coloring based on the relative fold-change, which was calculated against the respective negative control shRNA treatment type. On color scale, red demonstrates increase, and blue shows decrease in the expression level. Note that the color scale has been capped for both hippocampal and cortical results to ensure comparability (*i.e.*, fold-changes >5 in both heatmaps are shaded in the darkest *red* or *blue*).

In addition to the −4.8 kb enhancer region, MEF2 transcription factors have been shown to regulate *Bdnf* promoter I and IV (10, 15), but the contribution to all the major promoters of *Bdnf* has not been addressed. Therefore, next we investigated the role of different endogenous MEF2 transcription factors in the regulation of *Bdnf* transcripts comparatively in cultured cortical and hippocampal neurons. For this, we used the RNAi-mediated knockdown of *Mef2a*, *Mef2c* or *Mef2d* transcription factors. *Mef2b* was excluded as it has been shown to have low expression in cultured cortical and hippocampal neurons (6). Rat hippocampal (Fig. 3*A*) or cortical (Fig. 3*B*) neurons were transduced with lentiviruses encoding *Mef2a*, *Mef2c*, *Mef2d* or negative control shRNA. As the MEF2 transcription factors are known to function upon stimuli, *for example,* neuronal activity and after BDNF-TrkB-signaling (39), neurons were left untreated or treated for 3 h with KCl or BDNF to induce membrane depolarisation or TrkB-signaling, respectively.

First, we analyzed the efficiency of the knockdown tools by measuring the mRNA levels of *Mef2* family transcription factors. Silencing of *Mef2a*, *Mef2c* or *Mef2d* decreased the mRNA levels of all the respective *Mef2* family members in untreated as well as in KCl- or BDNF-treated hippocampal and cortical neurons. *Mef2a* and *Mef2c* shRNAs silenced the target genes with 80 to 90% efficiency (Fig. 3, *A* and *B*), silencing of *Mef2d* shRNA in untreated and treated cortical neurons was slightly weaker, resulting in 50 to 70% reduction of *Mef2d* mRNA (Fig. 3*B*).

Next, we studied the effect of MEF2-silencing on −4.8 kb eRNA expression (Fig. 3, *A* and *B*). In KCl-treated hippocampal neurons, silencing of *Mef2c* decreased the level of −4.8 kb eRNA ∼2.5-fold, while silencing of *Mef2d* increased −4.8 kb eRNA levels ∼2-fold (Fig. 3*A*). Surprisingly, silencing of *Mef2d* increased −4.8 kb eRNA levels ∼6-fold in BDNF-treated cortical neurons, while silencing of other *Mef2* family members had no effect on the level of −4.8 kb eRNAs in treated cortical neurons (Fig. 3*B*). These results indicate that MEF2D represses the −4.8 kb enhancer in cortical and hippocampal neurons, and that MEF2C acts as a transcriptional activator of this enhancer in KCl-treated hippocampal neurons.

Silencing of *Mef2a* had a tendency to decrease the levels of total *Bdnf* and almost all *Bdnf* transcripts in untreated and BDNF-treated hippocampal neurons (Fig. 3*A*), while silencing of *Mef2a* did not have any effect on the levels of *Bdnf* mRNA in cortical neurons (Fig. 3*B*). Silencing of *Mef2c* decreased the expression of *Bdnf* in untreated, KCl- and BDNF-treated hippocampal neurons, with the highest decrease, ∼6-7-fold, seen for *Bdnf* exon I-containing mRNA in untreated and KCl-treated neurons (Fig. 3*A*). Although silencing of *Mef2c* increased the levels of first cluster of *Bdnf* transcripts in KCl-treated cortical neurons, the expression levels of the second cluster of *Bdnf* transcripts and total *Bdnf* were decreased (Fig. 3*B*). Since *Bdnf* exon IV-containing transcripts are the major source of *Bdnf* mRNA in the cortical neurons (40), the small increase in the transcripts with exons from the upstream cluster did not influence significantly the total *Bdnf* mRNA levels. Silencing of *Mef2d* had the most drastic effects on the expression of *Bdnf*. In hippocampal neurons the *Bdnf* exon I-containing transcripts were upregulated ∼6-fold after KCl-treatment and *Bdnf* exon IV-containing transcripts were downregulated regardless of treatment (Fig. 3*A*). In cortical neurons the silencing of *Mef2d* led to significant upregulation of *Bdnf* expression both in untreated and BDNF-treated, but not in KCl-treated neurons (Fig. 3*B*).

Collectively, we determined that among the MEF2 family of transcription factors, MEF2C and MEF2D primarily govern the expression of *Bdnf* in both hippocampal and cortical neurons. Although we cannot definitively ascertain whether the observed effects of silencing are direct or indirect, our findings strongly suggest that the MEF2 family transcription factors have a pleiotropic role in the complex regulation of *Bdnf* gene, acting through both proximal and distal regulatory regions in distinct ways in cortical and hippocampal neurons.

## Discussion

In this study, we show how the family of MEF2 transcription factors exerts multifaceted control over the expression of its target gene *Bdnf.* This regulation involves distinct mechanisms operating in response to different stimuli and brain regions. While previous research has shown the −4.8 kb region to enhance *Bdnf* promoter I in heterologous context (6, 10), our findings show for the first time that in endogenous context the −4.8 kb enhancer regulates *Bdnf* in hippocampal neurons. Notably, we provide evidence of the −4.8 kb enhancer activity in rodents *in vivo* and describe the −4.8 kb enhancer in an open chromatin conformation in human hippocampal neurons. Finally, we determine that after silencing the MEF2 family members, MEF2C and MEF2D have the most significant impact on the transcriptional regulation of *Bdnf* in both cultured hippocampal and cortical neurons. Altogether, our results highlight the intricate interplay between MEF2 family transcription factors in the regulation of BDNF expression.

We demonstrate that the −4.8 kb enhancer regulates the first cluster of *Bdnf* transcripts, containing *Bdnf* exons I, IIc, and III, after neuronal activity in hippocampal neurons. The first cluster of *Bdnf* exons is known to exhibit a highly neuron-specific expression pattern and is suggested to be coregulated as a single cluster of promoters (18, 19, 20, 21). We have previously described an intronic enhancer region located downstream of the *Bdnf* exon III (+3 kb enhancer) which plays a crucial role in BDNF-TrkB signaling- and neuronal activity-induced expression of the first cluster of *Bdnf* transcripts in neurons (25). Although further studies are needed to determine the precise looping interactions, it is plausible that the −4.8 kb and +3 kb enhancer regions could synergistically facilitate the neuronal activity-dependent expression of the first cluster of *Bdnf* exons in hippocampal neurons. This aligns well with the hypothesis proposed in Tuvikene *et al.* 2021 (25), suggesting that the +3 kb enhancer functions as an anchor site for chromatin interactions between the *Bdnf* (promoters I, II, II) and the respective enhancers.

The hippocampus and cerebral cortex are the brain regions where BDNF is highly expressed (18, 19, 20). In addition to transcription factors involved in *Bdnf* regulation in both these brain regions (*e.g.*, CREB, NPAS4), numerous transcription factors regulate individual promoters of *Bdnf* depending on the brain region (16, 21). Although all *Bdnf* transcripts encode the same protein, it is known that transcript-specific modulation of *Bdnf* causes differential downstream cascades and phenotypes (41, 42). For example, in the hippocampus the upregulation of *Bdnf* exon I-containing transcripts increases the complexity of dendritic spine morphology, while upregulation of *Bdnf* exon IV-containing transcripts reduces fear expression (42). In contrast, mutations in *Bdnf* promoter IV impair the neuronal activity-dependent expression of BDNF important for the proper development of inhibitory synapses in the cerebral cortex (15). A differential engagement of enhancer regions could ensure this distinctive proportion of *Bdnf* transcripts in cortical and hippocampal neurons. Accordingly, the MEF2-dependent −4.8 kb enhancer is responsible for the increase in *Bdnf* exon I-containing transcripts upon neuronal activity and could thereby contribute to the complexity of dendritic spines in the hippocampus. Conversely, and as shown in the current study, the −4.8 kb enhancer is likely dispensable for the regulation of *Bdnf* exon IV-containing transcripts in cortical neurons, which comprise the vast majority of *Bdnf* transcripts needed there to refine synaptic connectivity. Moreover, the ability of MEF2 transcription factors to cooperate with cell type-specific transcription factors to activate tissue-specific enhancers (43) might explain the differential activation of regulatory regions crucial for BDNF expression in the cortex and hippocampus.

To date, the binding of MEF2 transcription factors has been linked to the −4.8 kb enhancer in hippocampus (10) and *Bdnf* promoter IV in cerebral cortex (6, 15, 16). The MEF2 transcription factor binding is not always restricted to the high affinity MRE site. For instance, MEF2 activates the transcription from *Bdnf* promoter IV through the CaRE1 element (6). In addition to proximal promoters, widespread binding of MEF2 transcription factors has been observed in distal regulatory regions (10, 43). Moreover, MEF2D binding to enhancers that lack the consensus MRE site has been shown to play a crucial role in photoreceptor cells, where MEF2D in cooperation with cell-specific transcription factors regulate the genes critical for photoreceptor functioning (43). Therefore, it is important to consider that our MEF2 knockdown results may arise from a combination of regulatory mechanisms, *i.e.*, binding of the MEF2 transcription factors both to proximal and distal regulatory regions of *Bdnf* gene and also to the regulatory regions of transcription factors regulating *Bdnf*.

While MEF2A, MEF2C, and MEF2D are widely expressed in the central nervous system, the proportion of each MEF2 family member varies significantly depending on the particular brain region (3, 6). For example, the expression of MEF2C in the hippocampus is highly restricted to the dentate gyrus (3, 44), where MEF2C is responsible for the development and morphology of dendritic spines (45, 46). Similarly, conditional deletion of MEF2C, but not MEF2A and MEF2D, is linked with altered memory formation and synaptic plasticity in the hippocampus (45, 47, 48), and MEF2C-related aberrations have been implicated in the development of autism-spectrum disorders (ASDs) (7). Recently it was shown that deletion of *Mef2c* specifically in adult-born dentate granule cells impaired contextual fear memory and caused deficits in social interactions, behavioral patterns associated with ASD (46). Although we have not addressed the exact region or subpopulation of neurons in the hippocampus in our studies, silencing of *Mef2c* had a profound effect, resulting in a significant reduction not only in the levels of the −4.8 kb eRNA but also in the overall expression of *Bdnf* mRNA in both basal levels and in KCl-treated hippocampal neurons. Further work on MEF2-regulated *Bdnf* expression in specific hippocampal subregions on single-cell level is needed. Nevertheless, our results describing *Bdnf* as a crucial downstream mediator of MEF2C could help to decipher the signaling cascade important for the proper synaptic development in the hippocampus.

Previous genome-wide studies have suggested that the redundancy and compensation among MEF2 family members can complicate the studies of MEF2 transcription factors (10, 48, 49). It is tempting to speculate that the observed effects on *Bdnf* regulation may also result from the intricate interplay between MEF2 family members. For example, when *Mef2d* is silenced, MEF2C might compensate for its absence, potentially enhancing the transcription of *Bdnf*. Studies in mouse cerebellar granule neurons have demonstrated that conditional MEF2D knockout induces compensatory genomic occupancy by MEF2A, but only at specific MEF2D sites (50). Although beyond the scope of this study, it would be interesting to study the endogenous binding of all the MEF2 transcription factors to *Bdnf* proximal and distal regulatory regions in wild-type and MEF2 knockout models in hippocampal and cortical neurons to confirm the direct participation in the transcriptional regulation.

From the MEF2 family members investigated in this study, silencing of *Mef2a* leads to the mildest effects when compared to the knockdown of *Mef2c* and *Mef2d*. It has been shown that brain-specific *Mef2a* knockout mice have no abnormalities, *Mef2a*-*Mef2d* double knockout mice have deficits in motor coordination but otherwise remain viable, and *Mef2a*-*Mef2c-Mef2d* triple knockout mice have decreased body weight together with neuronal apoptosis and postnatal lethality (48). Conversely, homozygous MEF2A knockout mice die during the first postnatal week due to the cardiac dysfunction (51), while MEF2D knockout animals appear to be vital and fertile, but exhibit problems in the circadian system as well as maturation and survival of photoreceptors (43, 52, 53). The described phenotypic peculiarities indicate that MEF2A might have a strong redundancy or it does not play a central role in the neural system, as seen for the regulation of *Bdnf* gene in cortical neurons.

In agreement with previous results (6), the silencing of *Mef2d* led to a significant increase in the mRNA levels of *Bdnf*. It is known that MEF2 transcription factors can, in addition to positive effects, repress their target genes (39). For example, in muscle cells, HDAC4 has been shown to form a repressor complex with MEF2D to control the regulation of genes (12, 13). Similarly, MEF2D in complex with HDAC4 could repress the expression of *Bdnf* gene in rat hippocampal and cortical neurons. We cannot rule out the possibility that MEF2D is crucial to refine the proper levels of BDNF, as abnormally increased levels of BDNF have been observed in the hippocampus and cortex of patients with temporal lobe epilepsy (54, 55). Whether the increase in *Bdnf* levels following the silencing of *Mef2d* indicates the effect of compensation or a way to refine the proper expression levels of BDNF, is a question that needs to be elucidated in the future.

In conclusion, the fine-tuning of *Bdnf* gene by MEF2 transcription factors illustrates distinct but robust regulatory mechanisms in the cortex and hippocampus. However, numerous exciting research questions are still awaiting exploration. For example, how do various MEF2 family members differentially control *Bdnf* transcription? How do the *Bdnf* promoters interact with the −4.8 kb enhancer and are the interactions different across distinct cell types? How do the outcomes of the current study relate to the regulation of *Bdnf* at the single cell level? Future studies of this intricate relationship will hopefully unlock important insights, clarifying not only the regulation of *Bdnf* gene but also adding new knowledge on the functioning of the brain.

## Experimental procedures

### Rat primary cortical and hippocampal neuron culture

For cultured neurons, all animal procedures were conducted in accordance with European Directive 2010/63/EU, reviewed and approved by the Ministry of Agriculture of Estonia (Permit Number: 45). Animals were maintained under a 12 h light/dark cycle in a humidity (50 ± 10%) and temperature (22 ± 1 °C) controlled room. Rats were group-housed (2–4 animals per cage), using either conventional polycarbonate or H-TEMP polysulfone cages with ad libitum access to water and food. The cultures of rat cortical and hippocampal neurons were generated from embryonic (E20/21) Sprague Dawley rats. Briefly, cortices and hippocampi were dissected and incubated in 1 ml of 0.25% trypsin and 1 mM EDTA solution (Gibco) for 10 min at 37 °C. Next, DNaseI (Roche) and MgSO_4_ (Sigma) were added to the trypsinized tissue to a final concentration of 0.5 mg/ml and 12 mM, respectively, and incubated for 10 min at 37 °C. Then, 275 μl of trypsin inhibitor (1%, Gibco), 110 μl of BSA (10%, Pan Biotech) and 50 μl of DNaseI (5 mg/ml, Roche) were added. The tissue was triturated and the resulting suspension was diluted in 1× Hank's balanced salt solution. The solution was centrifuged at 200*g* for 30 s. The supernatant was centrifuged again at 200*g* for 6 min. The supernatant was removed, and the precipitation was resuspended in prewarmed DMEM (Corning) containing 10% FBS (Pan Biotech). Plates were pre-treated in 0.1 M borate buffer with poly-L-lysine (0.2 mg/ml, Sigma Aldrich) for at least 1 h at room temperature. Cells were incubated for 2 h at 37 °C and 5% CO2. Then the medium was replaced with new cell medium (Neurobasal-A medium (NBA, Gibco) containing 1 × B27 supplement (Gibco), 100 U/ml penicillin, and 0.1 mg/ml streptomycin (Gibco) or 100 μg/ml primocin (Invivogen) and 1 mM L-glutamine (Gibco)). At 2 days *in vitro* (DIV), all of the medium was changed and 10 μM mitotic inhibitor 5′-fluoro-2′-deoxyuridine (Sigma-Aldrich) was added to inhibit proliferation of non-neuronal cells.

### Intracerebroventricular injection of kainic acid

Adult (3-month-old) male Wistar rats were used for intracerebroventricular injection (ICV) of kainic acid. The animals were housed in a specific pathogen-free environment, three per polypropylene cage in controlled temperature (23 ± 2 °C), humidity (50 ± 5%) and light (12 h light/dark cycle), with ad libitum access to food and water. The experiments were carried out in accordance with the National Institute of Health Guidelines for the Care and Use of Mammals in Neuroscience and Behavioral Research (The National Academics Press), European Communities Council Directive 2010/63/EU revising Directive 86/609/EEC, and national Decree-Law No 26 on March 4, 2014, and were approved by the local Animal Care Committee (OPBA) of University of Palermo, Italy and Ministry of Health, Italy. Up to 12 animals were randomly divided into four experimental groups: control (ICV-injected with saline solution), kainate 2 h, 4 h or 8 h (ICV-injected with kainate solution and sacrificed 2 h, 4 h or 8 h, respectively, after the onset of seizures). The surgical procedure was performed under aseptic conditions. Rats were initially anesthetized with intraperitoneal injection of chloral hydrate (200 mg/kg) and kept anesthetized for the entire duration of the surgical procedure with isoflurane. This procedure allows the early recovery of the animal from anesthesia immediately after the end of the surgery and the early and full manifestation of seizure behavior. Rats were placed in a David Kopf stereotaxic apparatus and received bilateral ICV injections of 0.35 μg/μl of kainate (Merck, dissolved in 0.9% physiological saline), using the following stereotaxic coordinates from the Bregma (according to Ref. (56)): AP  =  0, L  =  1.4, and V  =  4.3. The control group was ICV-injected with 1 μl of 0.9% physiological saline. Injections were performed by 30-gauge injector cannula that was connected by a piece of polyethylene tube to the 10 μl Hamilton syringe. Each injection was performed over 3 min, and following injection, the needle remained in the target location for 3 min to avoid kainate solution reflux along the needle tract and to achieve a proper diffusion of the drug. Animals were sacrificed by decapitation at different time points after the onset of epileptic seizures. Brain was quickly removed for dissection of hippocampus, frontal cortex, and cerebellum. Dissected brain areas were rapidly frozen and stored at −80 °C for later use.

### Lentivirus production and transduction

All used lentiviruses were produced in HEK293FT cell line (Thermo Fisher Scientific) on 145 mm cell culture dishes, cultured in DMEM (Pan Biotech) with 10% fetal bovine serum (FBS, Pan Biotech) and 1% penicillin-streptomycin (100 U/ml penicillin, 0.1 mg/ml streptomycin, Gibco) medium. The transfection solution contained 18.22 μg effector, 13.67 μg psPAX2 (Addgene) and 9.11 μg pVSVG (Invitrogen) plasmids with 2:1 PEI to DNA ratio. HEK293FT cells were transfected for ∼16 h at 37 °C and 5% CO2. After that, the transfection solution was replaced with 24 ml lentivirus production medium (DMEM (Pan Biotech), 10% FBS (Sera Plus), 1 mM pyruvate, 1× non-essential amino acids (Gibco), and 20 mM HEPES, pH 7.4). Medium containing lentivirus particles were collected twice with 24 h interval. To purify lentiviruses, the collected medium was centrifuged at 4500*g* for 5 min at +4 °C and the supernatant was filtered through a 0.45 μm polyvinylidene difluoride filter (Merck). 1/9 volume of Speedy Lentivirus Purification solution (Abm) was added to medium and lentiviral particles were precipitated with centrifugation at 7000*g* for 1 h at +4 °C. Lentiviral particles were resuspended in phosphate buffered saline (PBS) buffer.

To determine the relative lentivirus titres, neurons were infected with different dilutions of viruses at 0 DIV. At 7 DIV, 0.1 mg/ml DNasel (Roche) with 10 mM MgSO_4_ (Sigma) was added to neurons and incubated for 20 min at 37 °C to eliminate plasmid DNA contamination. Next, cells were lysed in proteinase K lysis buffer (0.08 mg/ml proteinase K, 30 mM Tris-HCl (pH 8,0), 1% Tween 20, 0.2% NP40, 1 mM EDTA). The lentiviral titers were analyzed based on provirus incorporation using provirus-specific Woodchuck Hepatitis virus post-transcriptional regulatory element or puromycin resistance (PURO) primers, and unrelated genomic region (for normalization (Supporting information). For functional experiments, equal amounts of lentiviral particles were used to transduce neurons, achieving >95% transduction efficiency. Rat cortical neurons were transduced at 0 DIV. At 7 DIV, tetrodotoxin was added to neurons to inhibit spontaneous neuronal activity. At 8 DIV, cells were treated with 25 mM KCl (PanReac AppliChem) and 5 μM D-(2R)-amino-5-phosphovaleric acid (D-APV, Cayman Chemical), with 50 ng/ml BDNF (Peprotech) or left untreated. After 3 h of treatment, neurons were lysed in 500 μl RLT buffer (Qiagen) containing 1% β-mercaptoethanol (ROTH).

### RNA isolation, cDNA synthesis and qPCR

Total RNA from rat cortical neurons or ICV-injected rat brain tissue was isolated using RNeasy Mini Kit (Qiagen) or with RNeasy lipid tissue kit (Qiagen), respectively, with on-column DNase digestion using RNase-free DNase set (Qiagen) according to the manufacturer’s instructions. RNA concentration was measured with a BioSpec-nano spectrophotometer (Shimadzu) or with a Nanodrop 2000c (Thermo Scientific) spectrophotometer. cDNA was synthesized from equal amounts of total RNA using Superscript III or IV Reverse Transcriptase (Thermo Fisher Scientific) with 1:1 mixture of oligo(dT)_20_ (Microsynth) and random hexamer primers (Microsynth). qPCR was performed in triplicates using 1 × HOT FIREpol EvaGreen qPCR Mix Plus (Solis Biodyne) or 1 × LightCycler 480 SYBR Green I Master (Roche) on LightCycler 480 II Real Time PCR instrument (Roche). All used qPCR primers are shown in Supporting information. *Hprt1* mRNA levels were used to normalize gene and enhancer RNA expression.

### DNA constructs

The RNA interference (pLKO.1-neg-shRNA, pLKO.1-MEF2A-shRNA, pLKO.1-MEF2C-shRNA, pLKO.1-MEF2D-shRNA) and luciferase plasmids (pGL3-BDNFpI-luc(-6143) and pGL3-BDNFpI-luc(-4495)) were kind gift from A. E. West, Department of Neurobiology, Duke University, North Carolina, United States of America. The gRNA targeting sequences (Supporting information) of the −4.8 kb enhancer were designed using Benchling CRISPR tool (http://www.benchling.com) as described previously in Avarlaid *et al.* 2024 (24), and cloned into the pRRL-U6-gRNA-hPGK-EGFP plasmid. Briefly, a total of 3 gRNAs targeting the ∼400 bp core region covering the −4.8 kb MRE sequence were used and the gRNA viruses were pooled for the CRISPR/dCas9 experiments. The plasmids (pLV-hUbC-dCas9-KRAB-T2A-GFP, pLV-hUbC-VP64-dCas9-VP64-T2A-GFP) used for CRISPR/dCas9-mediated modulation of the −4.8 kb enhancer were previously described in Tuvikene *et al.* 2021 (25).

### Transfection of cells and luciferase reporter assay

Rat cortical neurons were transfected at 5 DIV using Lipofectamine 2000 (Invitrogen) reagent. The transfection was carried out as duplicates in 48-well cell culture plates with each well containing 180 ng of luciferase reporter plasmid and 20 ng of pGL4.83-mPGK-hRluc normalizer plasmid in unsupplemented NBA with DNA to Lipofectamine ratio of 1:3. The transfection was carried out for 3 to 4 h and was terminated by changing the media back to the supplemented NBA that was previously collected from the neurons.

At 6 DIV, TTX (1 μM, Tocris Bioscience) was added to prevent spontaneous neuronal activity. At 7 DIV, neurons were treated with KCl (25 mM, PanReac AppliChem) and D-APV (5 μM, Cayman Chemical) for 6 h or left untreated. After treatments at 7 DIV the cortical and hippocampal neurons were lysed in 1 × Passive Lysis Buffer (Promega) and luciferase assay was performed using Dual-Glo Luciferase Assay (Promega) system. Luminescence signal was measured using GENios Pro Multifunction Microplate Reader (Tecan). For data analysis, background corrected Firefly luciferase signals were normalized with background corrected Renilla luciferase signals and the averages of duplicates were calculated.

### Statistical analysis

Sample size estimation was not performed, and randomization or blinding were not used. All tested hypotheses were specified before conducting the experiments. For cultured primary neurons, biological replicates were cultures obtained from rat pups of different litters. For *in vivo* experiments, biological replicates were individual animals. For statistical analysis, normalized data was log-transformed, mean centered, and autoscaled. The results were analyzed using Microsoft Excel 365 or RStudio version 2021.09.0 and R version R 4.1. Statistical significance was calculated using a two-tailed paired or unpaired *t* test, as indicated in figure legends, and *p*-values were not corrected for multiple comparisons. For graphical representation, data was backtransformed and error bars indicate upper and lower limits of backtransformed means ± SEM.

## Data availability

All data are contained within the article.

## Supporting information

This article contains supporting information.

## Conflict of interest

Jürgen Tuvikene and Tõnis Timmusk were employees of Protobios LLC. The authors declare no competing financial interests.
